# Dysphagia in Stroke: A New Solution

**DOI:** 10.4061/2010/570403

**Published:** 2010-06-30

**Authors:** Claire Langdon, David Blacker

**Affiliations:** Speech Pathology Department and Department of Neurology, Sir Charles Gairdner Hospital, Hospital Avenue Nedlands Western Australia 6009, Australia

## Abstract

Dysphagia is extremely common following stroke, affecting 13%–94% of acute stroke sufferers. It is associated with respiratory complications, increased risk of aspiration pneumonia, nutritional compromise and dehydration, and detracts from quality of life. While many stroke survivors experience a rapid return to normal swallowing function, this does not always happen. Current dysphagia treatment in Australia focuses upon prevention of aspiration via diet and fluid modifications, compensatory manoeuvres and positional changes, and exercises to rehabilitate paretic muscles. This article discusses a newer adjunctive treatment modality, neuromuscular electrical stimulation (NMES), and reviews the available literature on its efficacy as a therapy for dysphagia with particular emphasis on its use as a treatment for dysphagia in stroke. There is a good theoretical basis to support the use of NMES as an adjunctive therapy in dysphagia and there would appear to be a great need for further well-designed studies to accurately determine the safety and efficacy of this technique.

## 1. Introduction

Dysphagia (difficulty eating and swallowing) is extremely common following a stroke, affecting 13%–94% of acute stroke sufferers, with incidence relating to lesion size and location [1–3]. Dysphagia has been associated with higher rates of respiratory complications and increased risk of aspiration pneumonia [3–5], dehydration [6] and nutritional compromise [7]. It is also a socially penalising occurrence with a significant impact on sufferers' quality of life. While there is a rapid return to normal function for many stroke survivors, this is not always the case. Mann et al. found more than half of a group of stroke survivors admitted to hospital with dysphagia continued to demonstrate signs of swallowing impairment on videofluoroscopy when they were followed up at 6 months post stroke [8]. Dysphagia has been associated with poorer outcomes in stroke and increased likelihood of residential placement [7] and adds significantly to the estimated lifetime costs of between $12,031 and $73,542 [9] in Australian stroke survivors. 

Current treatment for dysphagia in Australia involves prevention of aspiration in the form of diet and fluid modifications, compensatory manoeuvres and positional changes, and rehabilitation exercises. This article discusses a newer treatment modality, neuromuscular electrical stimulation (NMES), and reviews the available literature on its efficacy as a therapy for dysphagia with a particular emphasis on its use as a therapy for dysphagia following stroke. 

## 2. What Is Neuromuscular Electrical Stimulation (NMES)?

Neuromuscular electrical stimulation has been utilised by physical therapists for several decades [10]. It allows bypass of the injured central circuitry to activate neural tissue and contract muscles to provide function to what is otherwise a nonfunctioning limb or structure [11]. NMES involves passing a small electrical current transcutaneously via electrodes to stimulate the neuromuscular junction and create a muscle contraction. It is only a viable therapeutic technique for muscles with an intact nerve supply, but has been used successfully on large skeletal muscles in many aetiologies, including stroke [11]. NMES for dysphagia involves applying electrodes to the muscles of the head and neck, and stimulating those muscles that are weakened or hemiparetic using pulses of electricity. This is generally combined with the subject swallowing food or fluids that are predetermined to represent the most appropriate consistency that the person can tolerate without aspiration. NMES is reported to have been used to treat Bell's Palsy [12], Opercular Syndrome [13], Multiple Sclerosis [14], head and neck cancer [15] and voice disorders [16], as well as stroke.

## 3. Reviewing the Literature about NMES as a Treatment in Dysphagia Therapy

The following review was based on a computer-assisted search using the database Medline. To identify relevant articles published between 2001 and 2010, the search strategy [(dysphagia) AND (neuromuscular electrical stimulation) OR (VitalStim)] was utilised. Only articles published in English and describing interventions with adults were included. This search identified eighteen articles discussing NMES published between 2001 and 2009. Of these, five were related to dysphagia as a consequence of stroke alone [17–21], eight to dysphagia relating to other aetiologies, including mixed aetiologies [14, 15, 22–27], three examined the effect of NMES on the swallowing mechanism in normal subjects [28–30], and two were related to NMES but in the form of a meta-analysis and a survey of users [31, 32].

## 4. NMES and VitalStim in Dysphagia Therapy

While the muscle stimulation units used by physiotherapists have been utilised in some studies of dysphagic patients [14, 20], a commercial device, the VitalStim unit, which was approved for use by the FDA in 2001 specifically for dysphagia rehabilitation has featured in the majority of studies in the literature. The manufacturers of the VitalStim unit state that on the basis of their FDA submission data, 97.8%–100% of patients treated will have improvement [26]. Research published in 2001 [17] reported that VitalStim was superior to thermal-tactile stimulation in treating a group of 99 acute stroke patients, however, this study has been widely criticised for methodological flaws. Despite these criticisms, VitalStim has been utilised by several thousand certified users in the USA without reports of adverse effects and with anecdotal reports of significant treatment successes. 

Over 9,000 clinicians in the USA have undergone training in the use of the VitalStim unit [31]. In 2007, results of a survey into use of NMES for dysphagia in the USA were published [32]. Survey respondents reported that stroke was the most commonly treated cause of dysphagia; outcomes were generally positive and no treatment-related complications had occurred following NMES treatment. Patients were treated in 3–5 sessions per week, usually for a duration of one-hour and reported above-average satisfaction with treatment outcomes. Survey respondents who were not using NMES reported that they were interested in the technique but sought published data on outcomes and safety.

## 5. Efficacy Data for NMES for Dysphagia 2001–2007

The developer of the VitalStim unit published data that examined the application of NMES on a group of 63 stroke patients and compared this to 36 stroke patients treated with thermal tactile stimulation. The study reported significantly greater improvement in the NMES group, but has been widely criticised for its many methodological flaws including use of a nonstandardised rating scale, utilisation of cricopharyngeal dilatation for some patients in the intervention group, and the use of a therapy technique (thermal tactile stimulation) that has not been demonstrated as efficacious [17].

In 2002, Leelamanit et al. published a study examining the use of NMES on 23 patients with dysphagia greater than 2 months' duration. All participants had dysphagia that was secondary to reduced laryngeal elevation and were treated with NMES for up to 4 hours daily. The authors reported that 20 of 23 showed improvement after a short period of “synchronized electrical stimulation” aimed at improving hyolaryngeal excursion. Duration of treatment ranged between 2 days to 30 days, with 6 of the 20 patients who demonstrated initial improvement requiring subsequent treatment due to relapse [22].

A study comparing VitalStim to “traditional” swallowing therapy techniques in 2006 reported on 22 subjects with dysphagia from mixed aetiologies (including stroke and head and neck cancer). Participants (*n* = 11) received VitalStim and their outcomes were compared to 11 subjects who received oromotor exercises, compensatory techniques, and thermal-tactile stimulation. Subjects in both groups demonstrated change: some improved and some demonstrated worse outcomes, although 9 of 11 subjects in the VitalStim group and 10 of 11 subjects in the control group were able to improve their diet consistencies postintervention. This study had several methodological shortcomings, including variability in the number and type of treatment sessions provided to subjects in the different groups, difference in time post stroke between commencing treatment and the small sample sizes [23]. 

Ludlow and colleagues examined 11 participants with chronic long standing dysphagia. They were interested in the effect of NMES on hyoid bone position with (1) no stimulation, (2) low sensory-level stimulation, and (3) maximally tolerated motor level stimulation when participants were (a) swallowing and (b) “at rest”. They reported that hyoid bone depression occurred with stimulation in the “at rest” condition in 9 of the 11 subjects and hypothesized that this downward movement of the hyoid would result in increased incidence of penetration and aspiration. They reported that no group change in aspiration was seen, in fact, participants who had the greatest downward hyoid movement with stimulation at rest had the greatest improvement during swallowing with the same degree of stimulation. The research team also noted improvements in airway protection for the group when they received low sensory levels of stimulation [24]. 

A retrospective analysis of 18 patients of mixed aetiologies treated using NMES reported that 50% of patients had improvement in their overall dysphagia score (*P* < .05); although none of the patients with severe dysphagia were able to discontinue enteral feedings [13]. The authors noted that the most significant gains were made by patients who were able to consume small amounts of food orally prior to treatment (*n* = 7). Following therapy, 6 of these 7 patients were able to discontinue tube feeding and two of them regained “normal” swallowing function [25]. The authors stated that NMES “is clearly a significant improvement over existing therapy in the treatment of dysphagia. Patients generally are very positive regarding its results,” page 43. It is interesting that the most significant gains in the study occurred for participants who were able to take small amounts of oral intake safely; presumably these patients had a basic swallow pattern established that could be improved upon by the VitalStim therapy. It should be noted that deconditioning from reduced muscle use may occur in dysphagia, with patients who are fed nonorally being especially susceptible to this phenomenon and reporting greater perceived effort in eating; this is particularly true for older patients with decreased functional reserve [33]. The phenomenon can best be summed up as “use it or lost it”.

Carnaby-Mann and Crary published results of treatment of six subjects with chronic dysphagia (ranging from 6 months to 15 years) treated with daily sessions of NMES to the anterior neck in a controlled experimental condition [26]. One patient withdrew from the study. The five patients who completed the study perceived significant improvement in their swallowing ability. Four of the five demonstrated clinically significant improvement in their ability. The remaining patient demonstrated improvement in score, but did not advance in dietary consumption to a point where this patient met a priori criteria for clinically meaningful change. Four of the five patients who completed the protocol were available for follow up at 6 months posttreatment: clinical benefits were maintained in these patients.

In 2007 a meta-analysis of available research into NMES was published [31]. This noted that although most of the published studies had reported positive results, many contained design flaws and threats to external validity including lack of objective measures of swallowing improvement and lack of controlled trials. A total of seven studies were included in the meta-analysis, with a total of 255 patients with dysphagia from multiple aetiologies (stroke, cancer, head trauma, and respiratory failure) and with mixed age and gender. One study had a 95% confidence interval that included an effect size of 0, consistent with no effect being demonstrated, while another had an effect size close to null. The remaining trials showed effect sizes over 0.4. Pooled results for the seven studies indicated a significant summary effect size, while analysis of change in dysphagia rating across the seven studies indicated a mean improvement of 20% in swallowing performance after treatment.

## 6. Effects of NMES on the Normal Swallowing Mechanism

Suiter et al. reported the effect of NMES on eight young adult subjects (mean age 27 for males and 25 for females) with normal swallowing function who received ten 1-hour treatment sessions with the VitalStim device. This study found no overall significant change in myoelectric muscle activity following treatment, although one subject demonstrated a large decrease and one a large increase in muscle activity following NMES. The authors commented that there was a need to determine optimal intensity of NMES treatment as higher intensities may be more effective at eliciting muscle contractions. They also noted that their protocol did not involve subjects actively swallowing which they conceded may have accounted for lack of change in myoelectrical activity [28].

Young normal subjects with and without electrical stimulation (at maximum tolerated stimulation levels) were examined on videofluoroscopy. These normal subjects showed significant hyolaryngeal depression with stimulation at rest, with reduced hyolaryngeal elevation during swallowing of a 5mL bolus. Swallows that occurred with stimulation were judged to be “less safe” than swallows without stimulation. The authors warned that because stimulation reduced hyolaryngeal excursion in normal volunteers, NMES would reduce elevation in dysphagia therapy [29]. Differences in hyoid bone movement between younger and older subjects without dysphagia have been reported in the literature, with hyoid elevating more slowly and remaining maximally elevated for a shorter duration in older subjects; however, the hyoid is noted to elevate farther, particularly for small bolus sizes [34].

## 7. Criticisms Regarding Use of NMES in Dysphagia Therapy

Logemann (2007) criticised VitalStim as it gave some clinicians an “easy out” from understanding a patient's underlying swallow physiology and stated that it had led to a large potential market “… for desperate patients willing to try anything”, page 11, and called for much more research to determine whether NMES has a role to play in the management of oropharyngeal swallowing disorders [35]. 

The New Zealand Speech-Language Therapists' Association published a position paper in 2007 that reviewed the literature on neuromuscular electrical stimulation published up to 2007. In its conclusion, the paper states “There is preliminary evidence that application of neuromuscular electrical stimulation in swallowing rehabilitation may ultimately present as a viable approach for swallowing impairment under some limited condition, however this information is not yet confirmed. Based on available published literature and the ethical guidelines that govern clinical practice, it is thus the position of the New Zealand Speech-Language Therapists Association that application of this treatment modality in swallowing rehabilitation cannot be supported by empirical evidence, has the under-evaluated potential to cause harm and does not meet the expectations for evidence-based practice. Application of this technique in the patient population is considered premature and should therefore not be utilized in the treatment of swallowing disorders until further evidence is available” [36].

Speech Pathology Australia produced a position statement in 2008 based on literature published to 2007 which stated “The current literature does not adequately address the benefits of the procedure nor its potential harm or long term effects”, page 3 [37]. A review article discussing NMES research published up to 2007 concluded that studies have provided promising findings, yet there is a need for higher quality controlled trials to provide evidence of the efficacy of NMES [38]. Since the Associations produced those papers, several new studies have been published; these are reviewed below.

## 8. Research Published Since 2007 on the Use of NMES to Alleviate Dysphagia Resulting from Stroke

Several European centres were involved in conducting a randomised trial of 25 patients with dysphagia that had persisted for more than 3 months following a hemispheric stroke Bülow et al. 2008 [18]. Twelve patients received NMES for one hour daily 5 days per week for 3 weeks. Thirteen patients underwent traditional swallowing therapy techniques of dietary modifications, positional techniques, or exercises to improve swallow function. Both groups demonstrated improvement posttreatment, leading the authors to conclude that “swallowing treatment will improve the awareness of how to eat and drink”, page 308. The authors cautioned that subjects' subjective feelings of improvement did not correlate with objective measures taken on videofluoroscopy, reporting that two subjects who received NMES required treatment for aspiration pneumonia after feeling that their swallowing difficulties had resolved and resuming normal diet and fluid intake.

In Thailand a single blind-controlled study on stroke patients with dysphagia persisting more than two weeks was reported, with 28 patients randomised to receive NMES (*n* = 15) or rehabilitation swallowing treatment (*n* = 13). Twenty-three patients completed the protocol and 21/23 patients demonstrated some improvement from pre- to posttherapy. Patients randomised to receive NMES had a significantly (*P* < .001) higher gain in their scores on the Functional Oral Intake Scale (FOIS), a 7-point ordinal scale that reflects the patient's ability to tolerate diet and fluids safely [19]. The mean duration post stroke was 23.18 (± 6.68 days) and 24.09 (± 6.61 days) in the rehabilitation versus NMES groups. While it can be argued that spontaneous recovery may be responsible for the changes seen in this study, the majority of patients who regain swallow function quickly post stroke tend to have this occur in the first two weeks [39, 40].

Lim et al. (2009) reported on 28 Korean stroke patients randomised to receive NMES plus thermal-tactile stimulation (*n* = 16) versus thermal-tactile stimulation (TTS) alone (*n* = 12). Six of 12 patients in the NMES group who were tube-fed were able to progress to oral feeding, compared to 1 of 7 in the TTS group. Other swallowing parameters (pharyngeal transit time, penetration and aspiration scores) and patient satisfaction ratings showed greater improvement in the NMES plus TTS condition compared to TTS alone. Unfortunately, the authors did not provide follow-up of their subjects, so it is unknown whether treatment gains were maintained [21]. 

Park et al. (2009) conducted a study into muscle activity that examined the effect of stimulation combined with a swallowing rehabilitation exercise over a period of two weeks of NMES with intensity set just above the sensory threshold. They found an increase in peak amplitude of sEMG immediately following treatment in six of eight subjects, but responses were not statistically significant. They also reported increased elevation of the hyoid bone following NMES therapy. For both peak amplitude and hyoid movement, subjects returned to baseline levels two weeks following treatment. Their study was conducted on young, healthy volunteers and the authors concede the difficulty in extrapolating their findings to dysphagic subjects [30].

Gallas et al. (2009) recruited 11 patients with chronic dysphagia as a result of stroke (hemispheric (*n* = 7) or brainstem (*n* = 4)) and treated them with electrical stimulation for 1 hour per day over 5 days. They reported improvement in overall swallowing function and decreased nutritional and respiratory consequences (*P* < .01). When evaluated using transcranial magnetic stimulation, motor cortical excitability and cortical mapping showed no modification following the electrical stimulation [20]. This is similar to the findings [35] examining the impact of NMES on hand function in stroke patients. This study found that NMES performed in an intensive manner (3–6 hours/day for 10 days over a 3-week period) produced significant improvements in functional activities but did not result in a change in number of voxels in any neuroanatomical area. Kimberley et al. have also noted that NMES has been seen to demonstrate the greatest amount of change in mild-to-moderately impaired subjects [41]. This may indicate that NMES has the greatest effect on muscles that have some volitional movement, where a response can be patterned in focused, intensive therapy efforts.

## 9. Future Directions

There is a great interest in NMES in Australia and there are currently a handful Australian Speech Pathologists who have undergone VitalStim certification. Some of these therapists are offering NMES treatment to patients in adult and paediatric settings. Its use is currently quite limited, however, with therapists constrained to using it in a research context by the Speech Pathology Australia Position Statement. 

Many of the studies that have been published into NMES have had small numbers and contained methodological flaws, been conducted on subjects with normal swallow function, or participants have been of a much younger age than the populations who experience the highest prevalence of dysphagia. Criticisms of the available studies have noted that there is investigator bias, lack of systematic application of techniques, lack of blinding, and many of the scoring systems that have been utilised as outcome measures have lacked validity and objectivity. There is also the question of relapse: many studies have failed to include a follow-up period that addresses whether patients will experience loss of function once the NMES therapy is withdrawn. However, despite these flaws, NMES seems to have some promise in the treatment of neurogenic dysphagia. Logemann suggested that in the rush to embrace a new treatment technique, proper scientific evaluation studies had been ignored. She commented that in order for a new technique to be introduced, there should be strong underlying neurophysiologic rationale for its application to an aetiology, followed by small group studies to define the efficacy of the procedure in a homogenous population. There should then be a move to studies of several larger groups with different diagnoses and finally randomised clinical trials should be conducted [35].

There is a good theoretical basis to support the use of NMES as an adjunctive therapy in dysphagia. The present data, upon which current guidelines are based, have many flaws, and there would appear to be a great need for further well-designed studies to accurately determine the safety and efficacy of this technique, the populations in whom it is most efficacious, and the most effective regime of treatment needed to produce and maintain results. It may be that the time has come to reexamine whether NMES is a useful adjunct to current dysphagia therapies, particularly in those patients with mild-to-moderate impairment whose swallowing difficulties have lasted longer than the first two weeks post-acute insult. This could take the form of an RCT if several stroke centres were prepared to contribute data in a collaborative study. The establishment of collaborative stroke networks in Australia paves the way for this. It is time now for clinicians with an interest in using NMES for dysphagia therapy to come together to further discuss a multicentre research trial.
